# Differential benefits of internet-based behavioral activation (iBA) for adolescents with high vs. low reward probability: a secondary analysis

**DOI:** 10.3389/fpsyg.2025.1444419

**Published:** 2025-09-23

**Authors:** Ye-Seul Kim, Eunbyeol Lee, Kee-Hong Choi

**Affiliations:** ^1^School of Psychology, Korea University, Seoul, Republic of Korea; ^2^KU Mind Health Institute, Korea University, Seoul, Republic of Korea; ^3^Mindeep Cognitive Behavioral Therapy Center, Seoul, Republic of Korea

**Keywords:** internet-based behavioral activation, depression, adolescents, reward probability, randomized controlled trial

## Abstract

**Introduction:**

Behavioral activation (BA) therapy is effective in treating depression in both adults and adolescents, and internet-based BA (iBA) therapy showed promise during the COVID-19 pandemic. Adolescents with depression often exhibit motivational impairment, which is associated with reduced motivation and engagement in rewarding activities as well as a heightened risk of problematic internet use. This study examined the differential benefits of iBA in alleviating depressive symptoms in adolescents with relatively high and low Reward Probability Index (RPI) levels.

**Methods:**

Using a randomized controlled trial design, this secondary analysis included 38 adolescents diagnosed with major depressive disorder, who were randomly assigned to either the iBA or internet-based psychoeducation and supportive therapy (iST) groups. Depressive symptoms, BA levels, and RPI were assessed at pre-treatment, post-treatment, and the 3-month follow-up.

**Results:**

The findings revealed that adolescents with low baseline RPI who received iBA showed a significantly greater reduction in depressive symptoms than those with high baseline RPI. Additionally, adolescents with low baseline RPI in the iBA group showed a significantly greater reduction in depressive symptoms than those in the iST group.

**Discussion:**

These results suggest that iBA is particularly beneficial for adolescents with low RPI, especially those experiencing anhedonia, and provides a promising approach to enhance treatment outcomes in this population. High accessibility of iBA may promote engagement in rewarding real-life activities.

## 1 Introduction

Depressive disorders often begin during adolescence and, if left untreated, can lead to persistent issues (Lewinsohn et al., 1994; Zisook et al., 2007). The global prevalence of adolescent depression has increased from 2011 to 2020, underscoring the severity of mental health issues (Shorey et al., 2022). Behavioral activation (BA) therapy is an effective psychotherapeutic approach for treating adult depression, and numerous studies support its efficacy. More recently, evidence-based research demonstrated its effectiveness in treating adolescents with depression as well (Dimidjian et al., 2006; Lee, 2023; Mazzucchelli et al., 2009). Recent circumstances during the COVID-19 pandemic have shown that internet-based behavioral activation (iBA) therapy is also effective for adolescents with depression (Lee, 2023). According to meta-analyses on the effectiveness of iBA for depression, iBA is effective in reducing depressive symptoms (Alber et al., 2023; Han and Kim, 2022).

Although the efficacy of iBA for adolescent depression has been established, the subgroups of adolescents that derive the most benefit from this intervention remain unclear. This study was conducted during the COVID-19 pandemic and aimed to determine the characteristics of adolescents who benefited the most from iBA. According to the response-contingent positive reinforcement (RCPR) framework, a lack of positive reinforcement can lead to reduced BA and the exacerbation of depressive symptoms (Carvalho et al., 2011; Dimidjian et al., 2011). The level of reward probability that an individual experiences is closely linked to depression. Reward probability refers to the likelihood of engaging in activities that can lead to rewards and has been identified as a crucial factor in the persistence of clinical depression (Carvalho et al., 2011).

The Reward Probability Index (RPI) is a vital measure that evaluates the difficulty of accessing environmental rewards and the ability to experience those rewards, thereby assessing the RCPR framework. As a measure, RPI is distinct from reward experience at the neurological level (Treadway and Zald, 2011) and differs from BA. Individuals with depression may have the capacity to engage in rewarding activities but may not experience the associated rewards due to low levels of BA (Hill et al., 2017). BA focuses on increasing opportunities for RCPR experiences through activity planning and targeting avoidance behaviors (Kennedy et al., 2024). Previous studies have indicated that low RPI is associated with the persistence of clinical depression, and higher RPI correlates with higher levels of reinforcement (Carvalho et al., 2011; Santos et al., 2017). Therefore, RPI is considered an important variable for explaining individual differences in treatment outcomes. This study aimed to examine whether the effectiveness of iBA varies with RPI levels.

Motivational impairment is a core deficit in individuals with major depressive disorder, and adolescents are particularly vulnerable to mental health issues such as depression due to the ongoing neurodevelopment of the neural reward system (Davey et al., 2008; Forbes, 2009; Yang et al., 2014). Previous studies have indicated that depressed adolescents exhibit reduced neurological responses to rewards compared to their non-depressed peers, leading to decreased efforts to obtain rewards (Forbes et al., 2020; Rzepa et al., 2017). Anhedonia in adolescents, characterized by a lack of motivation to pursue rewarding experiences, is associated with a low RPI (Forbes and Dahl, 2012). Consequently, adolescents with low RPI have fewer opportunities to gain rewards through positive experiences, which may increase the risk of problematic internet use as a compensatory strategy, thereby perpetuating anhedonia and depressive symptoms (Cheng et al., 2015; Shen et al., 2023).

From this perspective, BA therapy aims to increase positive reinforcement by enhancing participation in enjoyable experiences and activities, making it a promising approach for adolescents with low RPI (Forbes, 2009; McCauley et al., 2016). BA is rooted in the reinforcement theory of depression, which suggests that depressive behaviors result from the loss of RCPR (Lewinsohn, 1974). According to Manos et al. (2010) behavioral theory of depression, a decrease in goal-directed behavior leads to a reduction in environmental rewards, thereby increasing depression. Training individuals to engage consistently in activities that activate motivation during BA treatment can be clinically beneficial (Yang et al., 2014). Therefore, BA therapy offers a promising approach for adolescents with deficits in the reward processing circuits that are crucial for emotional regulation and motivation, making it particularly suitable for those with low RPI (Andersen and Teicher, 2008; Bertha and Balázs, 2013; Jacobson et al., 2006). BA increases access to goal-directed behavior, thereby reducing depression and enhancing the likelihood of rewards among depressed adolescents deficient in motivation. According to Hill et al. (2017), BA mediates the relationship between RPI and depressive symptoms. Building on these principles, iBA delivers the same therapeutic strategies online, thereby increasing accessibility for adolescents. Thus, iBA is expected to be beneficial for depressed adolescents with low pre-treatment RPI. However, there is a lack of research on the effectiveness of iBA in adolescents with low RPI.

### 1.1 Aims

This study primarily aimed to examine whether baseline RPI moderates the effect of treatment conditions (iBA vs. iST) on changes in depressive symptoms. Its secondary objective was to compare the baseline levels and changes in depressive symptoms, RPI, and BA between low- and high-RPI groups across treatment conditions. The study aimed to test the hypothesis that iBA is effective in improving depression in adolescents with low pre-treatment RPI.

## 2 Materials and methods

### 2.1 Participants

This study utilized a secondary analysis of data from Lee (2023), originally conducted for a doctoral dissertation titled “The Efficacy of Internet-based Behavioral Activation Treatment for Adolescents with Depressive Disorder.” The original study conducted by Lee (2023), encompassed 38 adolescents from middle and high schools. Detailed inclusion and exclusion criteria are described in the original study (see Lee, 2023), which included criteria such as age range (12–19 years), primary diagnosis of major depressive disorder, and agreement to participate in a two-arm parallel randomized controlled trial. Participants were randomly assigned in a 1:1 ratio using random numbers generated using Microsoft Excel. Exclusion criteria were established to ensure the safety of the adolescents. The study was approved by the Institutional Review Board of Korea University (KUIRB-2022-0361-07).

### 2.2 Procedure

**Figure 1:**
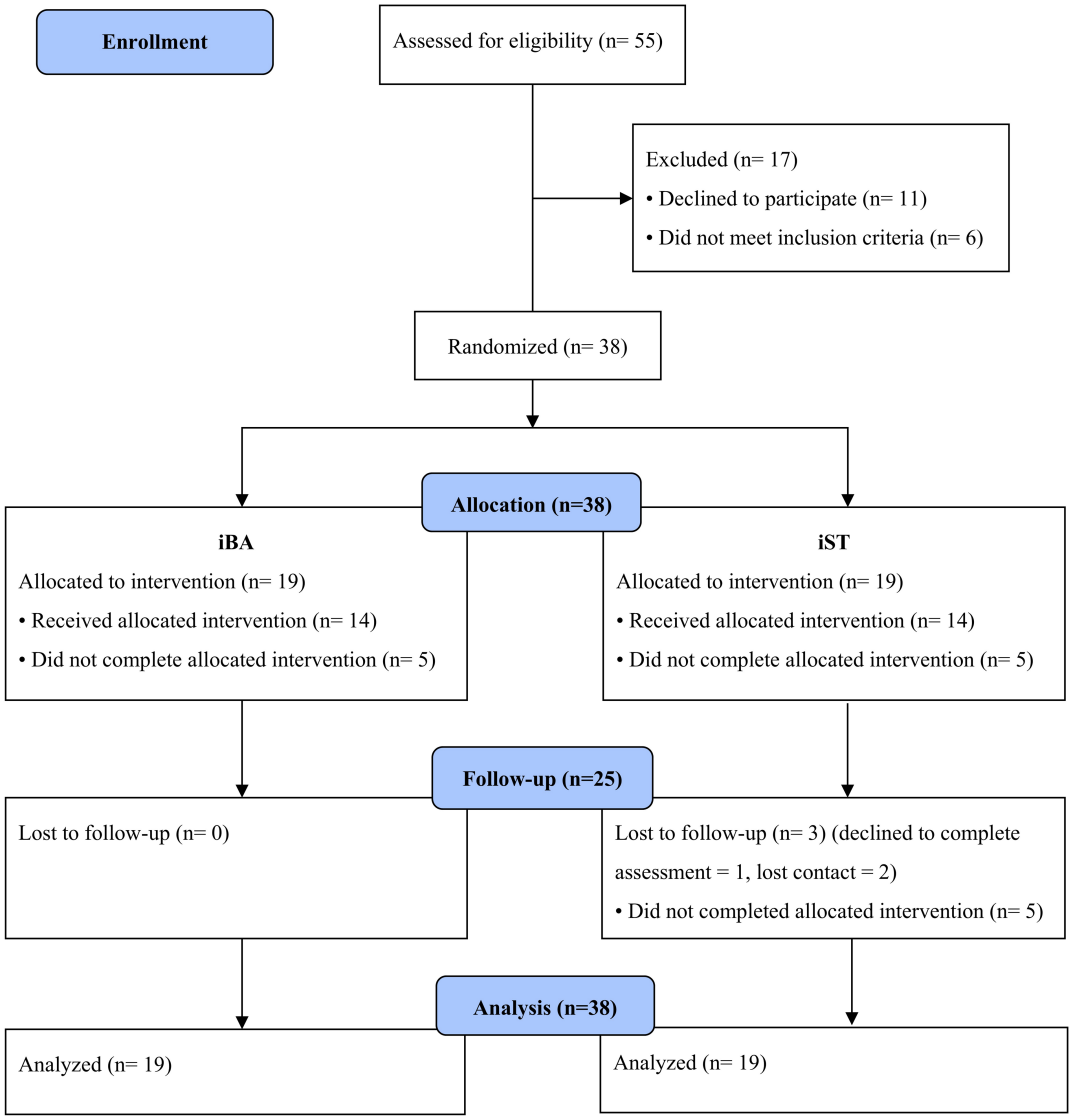
Flow chart of a randomized controlled trial (reproduced from Lee, 2023).

Participants were initially screened using the Mental Health Screening Tool for Depressive Disorders (MHS: D; Park et al., 2022) and the depression subscale scores of the Revised Child Anxiety and Depression Scale (RCADS; Chorpita et al., 2000). Of the 55 participants who met the initial screening criteria and consented to the baseline assessment, 11 declined to participate. Of the remaining 44 who completed the process, 6 did not meet the inclusion criteria, leaving 38 eligible for study participation (see Figure 1). These 38 participants were then randomly assigned to receive eight weekly sessions of either the individual iBA (*n* = 19) or iST (*n* = 19). The session structure and therapeutic components of each intervention are described in detail in Lee (2023). The reporting followed the relevant items from the CONSORT guidelines (Schulz et al., 2010; see Supplementary material 1).

### 2.3 Measures

#### 2.3.1 Korean children’s depression inventory 2nd edition

The Korean Children’s Depression Inventory 2nd Edition (K-CDI-2) is a 28-item self-report questionnaire that assesses depressive symptoms in children and adolescents. It is a Korean validation of the CDI-2, originally developed by Kovacs (1992). The K-CDI-2 provides scores for two scales (emotional and functional problems) and four subscales (negative mood/physical symptoms, negative self-esteem, inefficiency, and interpersonal problems). For Korean adolescents, a CDI score of 20 is the optimal screening cutoff (Bang et al., 2015). Cronbach’s alpha for internal consistency was reported as 0.85 for a sample of adolescents aged 13–17 years (Kim et al., 2018).

#### 2.3.2 Behavioral activation for depression scale

The Behavioral Activation for Depression Scale (BADS) is a 25-item self-report questionnaire designed to assess levels of activation and avoidance (Kanter et al., 2007). Items are rated on a scale of 0 to 6, with higher scores indicating higher BA levels. The BADS consists of four subscales: activation; avoidance and rumination; occupational and academic impairment; and social impairment. In Lee’s (2023) study, the subscale of occupational and academic impairment was renamed “academic impairment” to better reflect the developmental stages of adolescents. Cronbach’s alpha for the internal consistency of the BADS total score was 0.87 in a Korean validation study (Oh et al., 2017).

#### 2.3.3 Reward probability index

The RPI assesses response-contingent positive reinforcement through 20 items, divided into two factors. Eleven items measure reward probability, and nine items measure environmental suppressors. Response items are scored from 1 (strongly disagree) to 4 (strongly agree). Cronbach’s alpha was 0.88 at the initial administration and 0.92 at the retest, indicating high internal consistency (Carvalho et al., 2011).

### 2.4 Data analysis

This study utilized multilevel modeling (MLM) to compare the effects of iBA therapy and iST on changes in depressive symptoms among 38 adolescents with depression, based on baseline RPI. MLM is appropriate for longitudinal data analysis and allows for the inclusion of all participants in the analysis, including those who dropped out during treatment, to maximize data utilization (Ahn and Kwon, 2018; Mirman, 2017). A two-level modeling approach was used, with Level 1 representing time and Level 2 representing individual differences. At Level 1, changes in depressive symptoms over time were modeled, while at Level 2, individual differences, including treatment effects, baseline RPI and baseline BA levels were considered.

In this study, baseline BA levels were controlled to analyze the independent effect of initial RPI on changes in depression over time. To maintain consistency in comparisons between the two groups and facilitate model interpretation, grand mean centering was used for all variables (Raudenbush and Bryk, 2002). The RPI scores measured in advance were grand mean centered, and participants were categorized into groups with above-average and below-average RPI, which were then used as categorical moderator variables to compare the changes in depression over time between the two groups.

To compare whether the effect of baseline RPI on changes in depressive symptoms over time differed according to the treatment effect, a model that included the interaction of baseline RPI × time × treatment effect was constructed. Additionally, to control for the influence of baseline BA levels and baseline RPI, an MLM was modeled simultaneously including the interaction of baseline BA levels × time × treatment effect. Including these interactions ensured that the analysis accounted for the interrelated influences of baseline BA and RPI on treatment outcomes.

Statistical analyses were conducted using R software version 4.3.2. For multilevel analysis, the “lmerTest” (Kuznetsova et al., 2017) and “lme4” (Douglas et al., 2015) packages were utilized, and visualization of the multilevel analysis was performed using the “ggplot2” package (Wickham, 2011).

## 3 Results

### 3.1 Descriptive statistics

A total of 44 adolescents initially agreed to participate in the study, with 38 meeting the inclusion criteria; participants were randomly assigned to either the iBA group (*n* = 19) or the iST group (*n* = 19). The demographic and clinical characteristics of the participants were similar in both groups (Lee, 2023).

### 3.2 Changes in outcomes

Table 1 presents the mean changes in outcomes at pre-treatment, post-treatment, and the 3-month follow-up. In both the iBA and iST groups, participants with low baseline RPI had higher depressive symptoms and lower BA levels at baseline than those with high RPI. According to the K-CDI-2 results, in the iBA group, participants with high RPI showed a decrease from a baseline (*M* = 25.00) to follow-up (*M* = 18.25). In contrast, participants with low RPI in the iBA group had a higher baseline score (*M* = 33.20) but showed a more substantial reduction at follow-up (*M* = 12.67). This decrease was greater than that observed in the participants with low RPI in the iST group.

**Table 1:** Means and standard deviation at pre-treatment, post-treatment, and follow-up for the outcome variables.

Outcomes	Treatment	iBA		iST	
	Baseline RPI level	Low	High	Low	High
		M (SD)	M (SD)	M (SD)	M (SD)
K-CDI-2	Pre	33.20 (4.38)	25.00 (5.61)	32.75 (5.83)	27.64 (4.72)
	Post	17.33 (3.88)	19.38 (6.09)	22.00 (10.35)	21.89 (8.65)
	Follow-up	12.67 (5.89)	18.25 (7.89)	19.33 (9.61)	20.38 (7.35)
RPI	Pre	36.80 (1.81)	47.44 (6.39)	33.75 (4.83)	44.55 (2.66)
	Post	52.67 (5.57)	52.88 (5.72)	49.00 (7.68)	49.89 (8.57)
	Follow-up	57.33 (9.22)	53.25 (8.33)	48.00 (7.81)	47.75 (9.53)
BADS	Pre	52.70 (10.39)	71.00 (17.10)	49.13 (19.94)	75.82 (17.42)
	Post	103.33 (16.03)	85.37 (15.09)	85.40 (14.78)	92.56 (19.63)
	Follow-up	107.00 (19.22)	99.50 (14.76)	69.67 (12.01)	82.38 (28.04)

Additionally, in the iBA group, participants with low baseline RPI had a higher mean follow-up RPI score (*M* = 57.33) than those with a high baseline RPI (*M* = 53.25). For BADS, participants with low baseline RPI in the iBA group had a higher mean follow-up BADS score (*M* = 107.00) than those with high baseline RPI (*M* = 99.50). The increase in BADS mean scores from pre-treatment to follow-up for participants with low baseline RPI in the iBA group was 54.30 points, which was 33.76 points more than the increase in the iST group.

### 3.3 Multilevel analysis

The intent-to-treat MLM analysis revealed a significant decrease in depressive symptoms over time (*β* = −4.384, *p* = 0.013), indicating a significant reduction in depressive symptoms from pre-treatment to follow-up. Additionally, a significant interaction effect was found between treatment condition and time, indicating that individuals who received iBA treatment experienced significantly greater reductions in depressive symptoms over time compared to those who received iST treatment (*β* = −5.782, *p* = 0.011).

**Figure 2:**
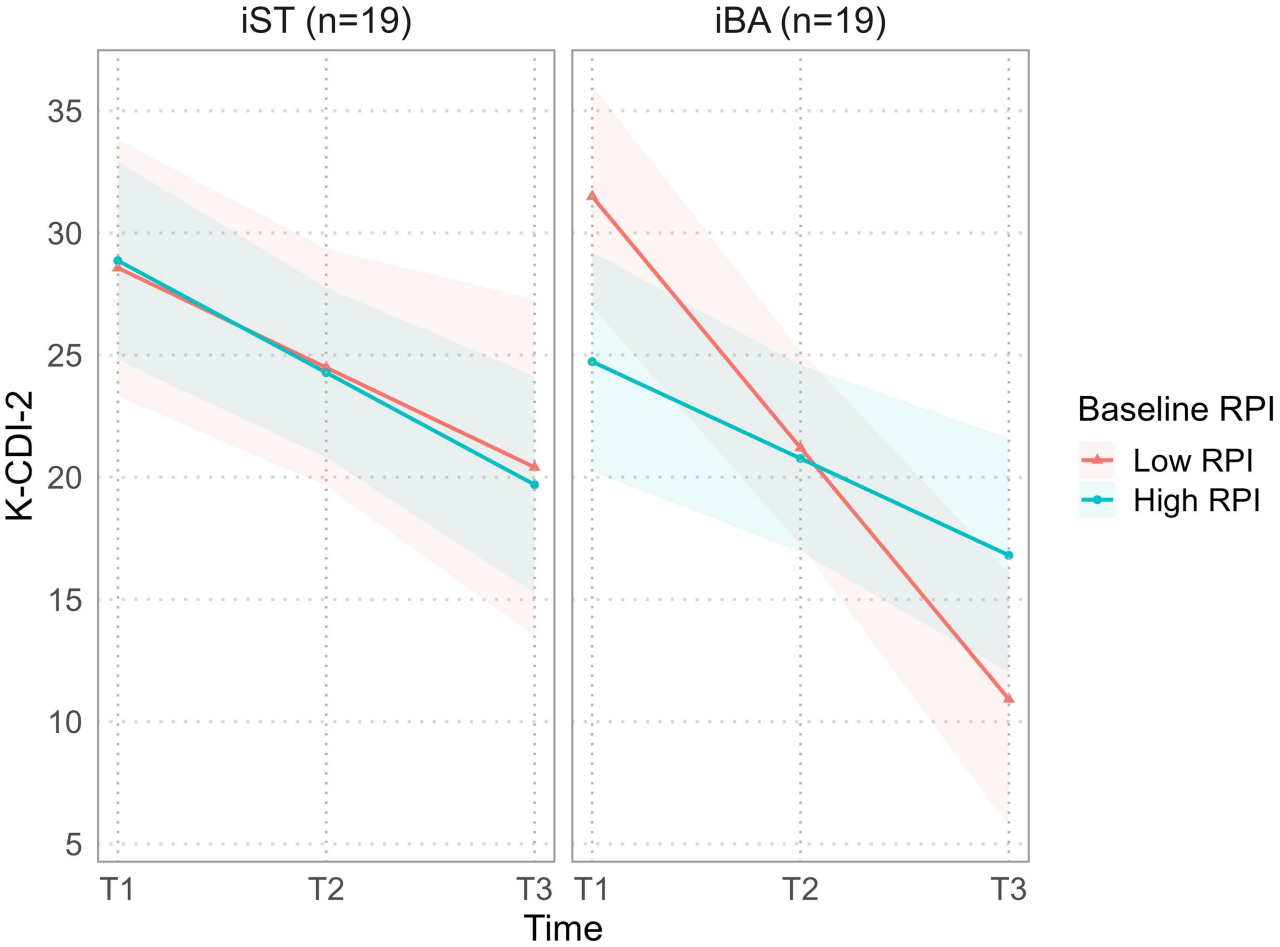
Plot of baseline RPI x time x treatment condition interactions from the model. The Baseline RPI × time interaction in the iST is shown in the left panel, and the baseline RPI × time interaction in the iBA is shown in the right panel. K-CDI-2; Korean Children’s Depression Inventory 2nd Edition.

Furthermore, there was a significant three-way interaction effect between baseline RPI, treatment condition, and time (*β* = 6.794, *p* = 0.026) presented in Figure 2. This suggests that adolescents with low baseline RPI who received iBA treatment showed a greater reduction in depressive symptoms over time than those with high baseline RPI. In other words, iBA treatment may be particularly effective in adolescents with low baseline RPI.

The baseline BA included as a covariate in the model showed a significant interaction with time, indicating that changes in depressive symptoms over time were influenced by the initial BA level (*β* = 0.157, *p* = 0.007). However, there was no significant difference in the interaction between baseline BA level and time across treatment condition (*β* = −0.087, *p* = 0.294). This suggests that the treatment effect of iBA is particularly effective for adolescents with low baseline RPI, independent of their initial BA levels.

## 4 Discussion

Anhedonia, a core feature of adolescent depression, is associated with impairments in reward processing and can lead to increased screen time and problematic internet use to compensate for diminished pleasure (Cangelosi et al., 2024; Christodoulou et al., 2020; Yu et al., 2023). However, such maladaptive behavioral patterns may exacerbate depressive symptoms and anhedonia while reducing opportunities for positive reward experiences in real life, thereby perpetuating a vicious cycle (Barkus, 2021; Ellis et al., 2019; Rzepa and McCabe, 2019; Shen et al., 2023). Moreover, during the COVID-19 pandemic, the risk of sustaining this vicious cycle between adolescent depression, anhedonia, and problematic internet use was heightened due to restrictions on in-person interactions (Cai et al., 2023), further limiting opportunities for adolescents with anhedonia to experience rewards. Because iBA is delivered remotely, it can improve treatment accessibility for adolescents with low motivation and limited ability to experience rewards (e.g., low RPI). Targeting avoidance and enhancing intrinsic motivation may help reduce problematic internet use and increase opportunities for rewarding, real-life experiences.

In light of this, the present study examined changes in depression based on baseline RPI across different treatment conditions to determine which characteristics of adolescents benefit more from iBA. As a measure, RPI differs from reward sensitivity, as the latter refers to neurological responses to rewards and indicates the intensity of the reaction to receiving a reward. By contrast, RPI refers to the likelihood of engaging in activities that can lead to rewards, making these concepts related but distinct (Treadway and Zald, 2011). Low levels of RCPR are associated with the persistence of clinical depression, and environmental rewards play a crucial role in reducing depression (Collado et al., 2014; Santos et al., 2017). From a behavioral perspective, low RCPR is linked to avoidance behaviors (Wagener and Blairy, 2015). The iBA in this study focused on addressing avoidance behaviors and increasing access to positive experiences to enhance positive reinforcement (Lee, 2023).

The results indicated that adolescents with low RPI experienced a more significant reduction in depressive symptoms in the iBA group than in the iST group. This suggests that iBA effectively reduced depressive symptoms in adolescents with low RPI by creating a rewarding environment. These findings support previous research indicating that short-term rewards through behavioral reinforcement are effective for individuals with low RPI (Jacobson et al., 2006; Khazanov et al., 2022) and confirm the relationship between increased behavioral activation and depression reduction (Collado et al., 2014; Yamamoto et al., 2019).

Aoki et al. (2021) showed that low goal-directed behavior reduces environmental rewards, leading to increased anhedonia. Similarly, this study found that iBA effectively increased environmental rewards and alleviated depressive symptoms, particularly in adolescents with lower RPI. This suggests that iBA may help restore impaired reward processing, consistent with Hanuka et al. (2023), who found that an internet-based cognitive behavioral therapy program improved reward circuit connectivity and reduced anhedonia compared with a control group.

These results emphasize the importance of reward, a core element of behavioral theories (Lewinsohn et al., 1980; Takagaki et al., 2016). Carvalho and Hopko (2011) reported that RPI significantly mediates depression caused by cognitive and behavioral avoidance, supporting the effectiveness of iBA in reducing avoidance and enhancing positive experiences.

Furthermore, the results of this study align with those of previous research, indicating that iBA, which increases access to rewards, is particularly effective for adolescents with low RPI (Collado et al., 2014; Santos et al., 2017). Elements of iBA, such as monitoring daily activities to allocate more attention to recent meaningful or enjoyable activities, and planning for future rewarding activities, likely enhance expectations and motivation for rewards in adolescents with low RPI (Lee et al., 2016; Treadway and Zald, 2011; Lee, 2023).

Given that adolescence is a period of neural development in the reward system, approaches that enhance RPI can play a crucial role in preventing and treating depression (Forbes, 2009; Yang et al., 2014). Moreover, because anhedonia can negatively affect adolescents’ social interactions and internet use, the high therapeutic accessibility of iBA may serve as a valuable early intervention to mitigate the detrimental impact of problematic internet use.

This study is significant in several ways. First, it longitudinally confirmed the benefits of iBA in adolescents with low RPI, which is an important clinical finding. Second, it highlights the potential of approaches that strengthen the reward system to improve treatment outcomes for depressed adolescents (Collado et al., 2014; Yamamoto et al., 2019). Enhancing RPI is particularly crucial during adolescence when the reward system is developing, making this approach vital for the prevention and treatment of depression (Forbes, 2009; Yang et al., 2014).

## 5 Limitations

This study had several limitations. First, the small sample size limited the generalizability of the results. Future studies should include a larger sample size to increase the reliability of the findings. Second, dividing the RPI levels based on the sample mean may have introduced bias in interpreting the results, limiting the generalizability of the findings. The average RPI for the high-RPI group in this study corresponds to the average level of depressed individuals in other studies, making generalization difficult. Finally, the RPI, as being a self-reported measure, does not reflect actual behavior, thus limiting its ability to accurately assess RCPR. Despite these limitations, this study provides important insights into the benefits of iBA for adolescents with depression and suggests that future research can build on these findings by addressing the limitations of producing more robust and reliable results.

## 6 Conclusion

This study explored the different benefits of iBA in adolescents with varying baseline RPI levels. The results show that iBA reduces depressive symptoms among adolescents with low RPI. By enhancing access to positive experiences and addressing avoidance behaviors, iBA significantly improved outcomes compared with iST. These findings emphasize the importance of personalized treatment approaches, considering individual differences in RPI, and have clinical implications for the applicability of iBA for anhedonia. Given its high accessibility, iBA may also help shift adolescents from problematic internet use to more rewarding real-life activities. Future research should include larger samples and refine the assessment methods to further validate and generalize these results. Despite these limitations, this study provides crucial insights into the optimization of iBA for adolescents with depression.

## Data Availability

The original contributions presented in the study are included in the article/Supplementary material, further inquiries can be directed to the corresponding author.
